# Effects of Remote Ischemic Preconditioning on Heme Oxygenase-1 Expression and Cutaneous Wound Repair

**DOI:** 10.3390/ijms18020438

**Published:** 2017-02-17

**Authors:** Niels A. J. Cremers, Kimberley E. Wever, Ronald J. Wong, René E. M. van Rheden, Eline A. Vermeij, Gooitzen M. van Dam, Carine E. Carels, Ditte M. S. Lundvig, Frank A. D. T. G. Wagener

**Affiliations:** 1 Department of Orthodontics and Craniofacial Biology, Radboud University Medical Center, Nijmegen 6500HB, The Netherlands; niels.cremers@radboudumc.nl (N.A.J.C.); rene.vanrheden@radboudumc.nl (R.E.M.v.R.); carine.carels@radboudumc.nl (C.E.C.); dittelundvig@hotmail.com (D.M.S.L.); 2 Department of Rheumatology, Radboud University Medical Center, Nijmegen 6500HB, The Netherlands; eline.vermeij@radboudumc.nl; 3 Central Animal Laboratory, Radboud University Medical Center, Nijmegen 6500HB, The Netherlands; kim.wever@radboudumc.nl; 4 Radboud Institute for Molecular Life Sciences, Nijmegen 6500HB, The Netherlands; 5 Radboud Institute for Health Sciences, Nijmegen 6500HB, The Netherlands; 6 Department of Pediatrics, Stanford University School of Medicine, Stanford, CA 94305, USA; rjwong@stanford.edu; 7 Department of Surgery, University Medical Center Groningen, Groningen 9700RB, The Netherlands; g.m.van.dam@umcg.nl; 8 Department of Human Genetics, Radboud University Medical Center, Nijmegen 6500HB, The Netherlands; 9 Department of Oral Health Sciences, Faculty of Medicine, KU Leuven, 3000 Leuven, Belgium

**Keywords:** remote ischemic preconditioning, heme oxygenase-1, tissue injury, wound repair

## Abstract

Skin wounds may lead to scar formation and impaired functionality. Remote ischemic preconditioning (RIPC) can induce the anti-inflammatory enzyme heme oxygenase-1 (HO-1) and protect against tissue injury. We aim to improve cutaneous wound repair by RIPC treatment via induction of HO-1. RIPC was applied to HO-1-*luc* transgenic mice and HO-1 promoter activity and mRNA expression in skin and several other organs were determined in real-time. In parallel, RIPC was applied directly or 24h prior to excisional wounding in mice to investigate the early and late protective effects of RIPC on cutaneous wound repair, respectively. HO-1 promoter activity was significantly induced on the dorsal side and locally in the kidneys following RIPC treatment. Next, we investigated the origin of this RIPC-induced HO-1 promoter activity and demonstrated increased mRNA in the ligated muscle, heart and kidneys, but not in the skin. RIPC did not change HO-1 mRNA and protein levels in the wound 7 days after cutaneous injury. Both early and late RIPC did not accelerate wound closure nor affect collagen deposition. RIPC induces HO-1 expression in several organs, but not the skin, and did not improve excisional wound repair, suggesting that the skin is insensitive to RIPC-mediated protection.

## 1. Introduction

Severe skin wounds following burns, trauma, or surgery often lead to scar formation and impaired functionality [1]. Cutaneous wound repair is a dynamic and highly regulated process, involving several overlapping phases: inflammation, proliferation, and remodelling [2]. Aberrant wound repair and scarring occurs following prolongation of the inflammatory phase that together with oxidative stress fuels (myo)fibroblast proliferation and interferes with myofibroblast apoptosis [2]. This leads to excessive deposition of extracellular matrix proteins, subsequently promoting excessive scar formation [3,4]. Unfortunately, conventional therapies to accelerate wound repair and to prevent scarring are insufficient [5,6,7]. Therefore, adjuvant therapies aimed at resolving inflammation are warranted. Pharmacological preconditioning has been shown to improve wound repair, as exemplified by heme and curcumin that also induce the cytoprotective protein heme oxygenase-1 (HO-1) [8,9,10,11,12,13,14]. HO-1 is one of the most important enzymes protecting against oxidative and inflammatory insults [15]. HO catabolizes heme to biliverdin, free iron, and carbon monoxide (CO). Biliverdin is then rapidly converted to the antioxidant bilirubin by biliverdin reductase [16,17]. The iron scavenger ferritin is co-induced by HO-1 and renders iron inactive [18]. Recent studies have shown that induction of HO-1 expression attenuates the inflammatory response and accelerates wound healing in HO-1-deficient mice; whereas, decreased HO activity in mice results in slower cutaneous wound closure [9,19]. In addition, intraperitoneal administration of the HO-effector molecule bilirubin accelerates wound repair [20]. Since increased HO-1 expression improves wound repair, its induction may be a good candidate for preventing aberrant cutaneous wound repair.

A promising novel preconditioning strategy is ischemic preconditioning (IPC), hereby, short cycles of ischemia/reperfusion to an organ protects against subsequent more harmful insults to the same organ. In remote ischemic preconditioning (RIPC), the target organ is not subjected to the initial stress, but a remote organ, e.g., the hind limb, is exposed. [21,22]. Interestingly, RIPC protects against injury in the liver [23,24,25,26], lung [27], intestines [28], heart [29,30], and kidneys [31,32] often via the induction of HO-1, since inhibition of HO-activity abrogates the protective effects of RIPC [23,26,33]. Following RIPC, there exists both a rapid phase of protection initiated within 1h after preconditioning, and a later phase after one to several days [21,34]. In addition, different modes of action have been reported between single and repeated RIPC procedures, as demonstrated by differential expression of genes involved in autophagy, endoplasmic reticulum stress, mitochondrial oxidative metabolism, and cell survival [35,36].

Successful translation towards its clinical use was recently established by inducing temporary occlusion and restoration of blood flow in arm or thigh of patients [29,30,37]. Patient outcome after myocardial surgery was significantly improved when RIPC was applied before surgery [27]. However, recently conflicting results have been reported showing that RIPC does not always mediate protection [38,39,40,41]. Data from animal and human studies demonstrated the need for careful interpretation because of translational differences [38,39,40,41,42,43]. RIPC improves microcirculation by an increase in tissue oxygenation and capillary blood flow in the skin [44] and skin flaps [45], and forms a novel target for skin flap transplantation [46] and the healing of diabetic foot ulcers [47,48]. Although RIPC has been shown to protect in several models of tissue injury, its role in cutaneous excisional wound healing is still unclear. We postulated that RIPC induces HO-1 expression and improves skin repair following excisional skin injury.

## 2. Results

### 2.1. Effects of RIPC on HO-1 Promoter Activity and HO-1 mRNA Expression in Mice

**Figure 1:**
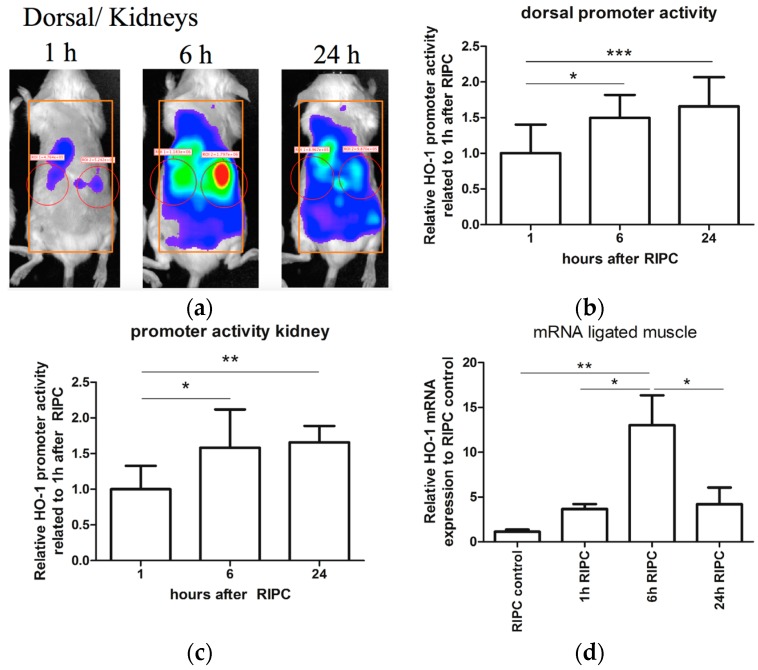

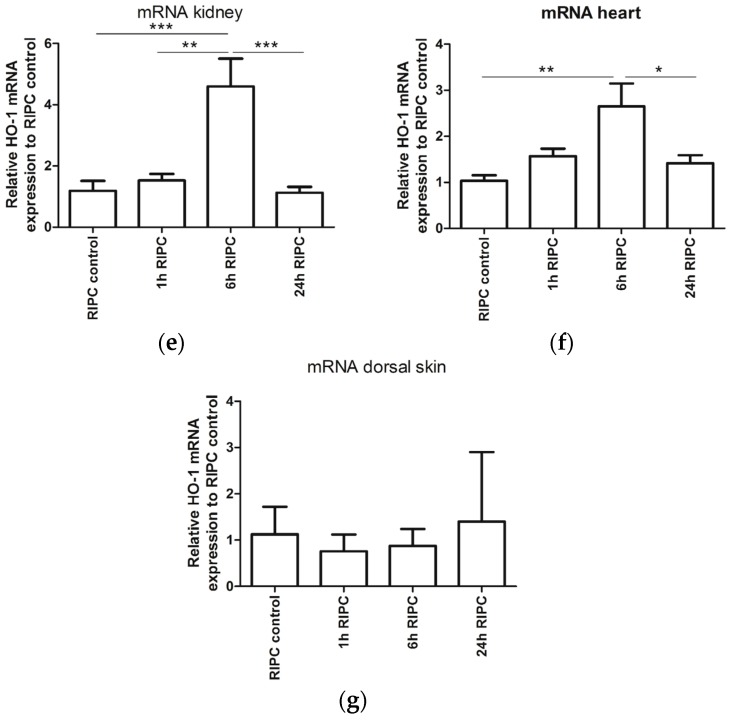
Heme oxygenase-1 (HO-1) promoter activity (**a**–**b**) and mRNA expression (**b**–**g**) after remote ischemic preconditioning (RIPC) treatment. (**a**) Representative dorsal images of HO-1 promoter activity after RIPC over time. Both the overall dorsal side (inserted orange rectangles) and the specific regions of the kidneys (inserted red circles) were analyzed and the total flux of emitted photons per second was quantified. Quantification of HO-1 promoter activity in the overall dorsal area after RIPC treatment (**b**); and locally in the regions of the kidneys (**c**), and HO-1 mRNA expression in muscle at the place of ligation (**d**); kidney (**e**); heart (**f**); and dorsal skin (**g**), 1, 6 and 24 h after RIPC treatment compared to untreated controls (*n* = 6 animals per group). Data are expressed as mean ± SD of six individual mice. * *p* < 0.05, ** *p* < 0.01, *** *p* < 0.001.

**Figure A1:**
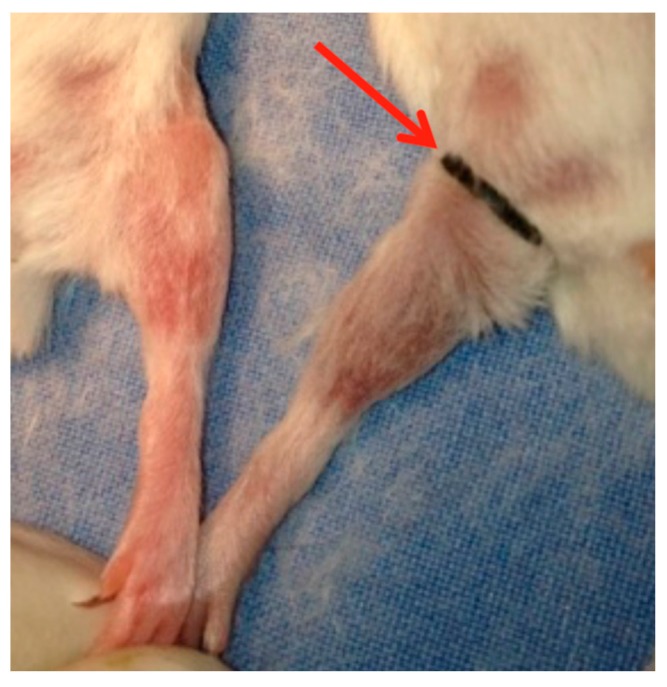
Hind limb ischemia by ligation using elastic ring (**red arrow**). Note the difference in the color of the legs after obstruction of the blood flow, confirming RIPC treatment was successful.

RIPC can induce HO-1 expression in different organs. In order to evaluate if RIPC can induce HO-1 expression, we used a combination of HO-1 promoter activity and HO-1 mRNA analyses in HO-1-*luc* transgenic (Tg) mice. We previously demonstrated that treatment with cadmium chloride (CdCl_2_) potently induced HO-1 promoter activity in the liver and kidney using the HO-1-*luc* Tg model [49]. To validate the RIPC model, we corroborated that blood flow was indeed hampered after applying elastic rings (Figure A1). After RIPC treatment, HO-1 promoter activity was measured using the In Vivo Imaging System at 1, 6, and 24 h. Measurements of HO-1 promoter activity were acquired at the dorsal aspect of each mouse (Figure 1a). Because of variations in HO-1 promoter activity, each mouse served as its own control. Relative HO-1 promoter activity of the dorsal side of the mice after RIPC treatment is shown over time (Figure 1b). We found a significant increase in HO-1 promoter activity after 6 and 24 h of RIPC treatment compared to 1 h after RIPC. HO-1 promoter activity was strongly observed in the renal area, suggesting RIPC induced HO-1 promoter activity in an organ-specific manner 6 and 24 h after RIPC treatment when compared to 1 h after RIPC treatment (Figure 1c).

To discriminate whether the skin or underlying organs were responsible for the increase in HO-1 promoter activity, and to test whether RIPC induced HO-1 expression in a tissue-specific manner, we measured HO-1 mRNA expression levels in the skin and several organs 1, 6 and 24 h after RIPC using RT-PCR and then compared these results to untreated controls. First, HO-1 mRNA expression in the hind limb muscles that had been exposed to repeated ischemia/ reperfusion cycles were measured and significantly increased 6 h after RIPC treatment when compared to untreated controls and other time points after RIPC (Figure 1d). We then analyzed the effects of RIPC on HO-1 mRNA levels in remote organs like kidney, heart, and skin. We also observed a significant increase in HO-1 mRNA expression in the kidney 6h after RIPC treatment when compared to untreated controls and other time points after RIPC (Figure 1e). Similarly, in the heart, HO-1 mRNA levels significantly increased 6h after RIPC treatment when compared to untreated controls (Figure 1f). No significant induction was found at other time points compared to untreated controls. After 24 h, HO-1 mRNA levels returned to control levels. However, no significant induction in HO-1 mRNA expression was detected in the skin at any time point (Figure 1g). In conclusion, we demonstrated a tissue- and time-specific induction of HO-1 promoter activity and HO-1 mRNA expression after RIPC treatment.

### 2.2. Effects of Early or Late RIPC on Excisional Cutaneous Wound Closure in Mice

**Figure 2:**
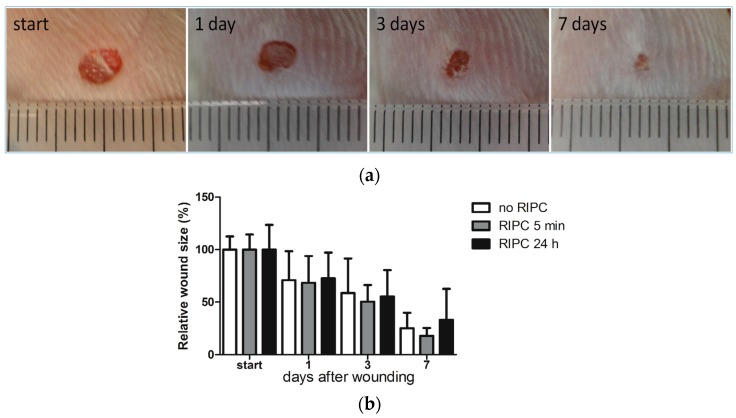
Excisional wound closure in time after RIPC treatment 24 h and directly after wounding compared to the control group. Representative images of the wounds of a single mouse receiving no RIPC treatment at day 0, and at 1, 3, and 7 days after wounding (ruler is incorporated in the pictures and each bar represent 1 mm) (**a**) and their relative wound sizes after different treatments in time, compared to control group at day 0 (**b**). Data are expressed as mean ± SD. No significant differences were observed between the different groups: no RIPC (*n* = 8), RIPC 5 min (*n* = 6) and RIPC 24 h (*n* = 6).

Since RIPC was observed to improve the cutaneous microcirculation [44], we next investigated whether RIPC could also modulate cutaneous wound repair. RIPC induced HO-1 expression in several organs, but not in the skin. Interestingly, HO-1 can also promote regeneration in a paracrine fashion via its versatile effector molecules biliverdin/bilirubin, CO, and ferritin [20,50,51,52,53,54], which are increased following (R)IPC [55,56,57,58]. Moreover, RIPC-mediated protection can act via various alternative signaling pathways, including humoral, neuronal, and systemic mechanisms [22,59]. Since there are early and late protective effects of RIPC, we evaluated whether early (5 min) and/or late (24 h) RIPC treatment before wounding improved full-thickness excisional wound closure. Examples of untreated excisional wounds are shown in Figure 2a. Wound sizes were normalized to the wound size at day 0 (Figure 2b). As expected, after quantification of the wound surface area, we found a reduction in the wound size over time. However, no significant differences were observed in wound closures between early or late RIPC treatment mice and controls.

### 2.3. HO-1 mRNA and Protein Expression in Wounds After RIPC

**Figure 3:**
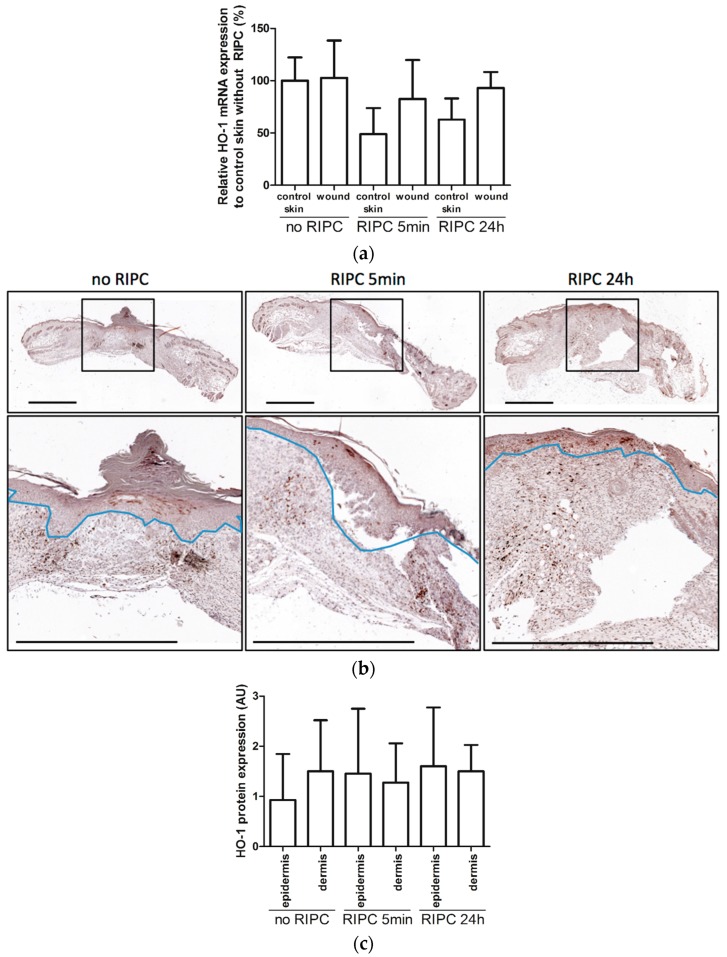
HO-1 expression in wounds. (**a**). HO-1 mRNA expression in unwounded (control) skin at day 0 and wounds after 7 days for the different treatments compared to control skin; (**b**) HO-1 protein expression in control, and early and late RIPC-treated wounds after 7 days of healing. Region above the marked blue line is the epidermis and underneath the blue line is the dermal layer (bars represent 1 mm); (**c**) Scored HO-1 protein staining in epidermis and dermis of the wounds after 7 days in arbitrary units (AU). Data are expressed as mean ± SD. No significant differences were observed between the different groups: no RIPC (*n* = 8), RIPC 5 min (*n* = 6) and RIPC 24 h (*n* = 6).

To further elucidate the role of RIPC-induced HO-1 expression during wound repair, we investigated whether RIPC modulates HO-1 mRNA and protein expression in day-7 wounds. Using RT-PCR, HO-1 mRNA expression was assessed in the wounds and compared to non-wounded day-0 skin (Figure 3a). Here, we found no significant differences between the wounds and their corresponding control skins for both RIPC-treated and control groups as well as between the different treatment groups.

**Figure A2:**
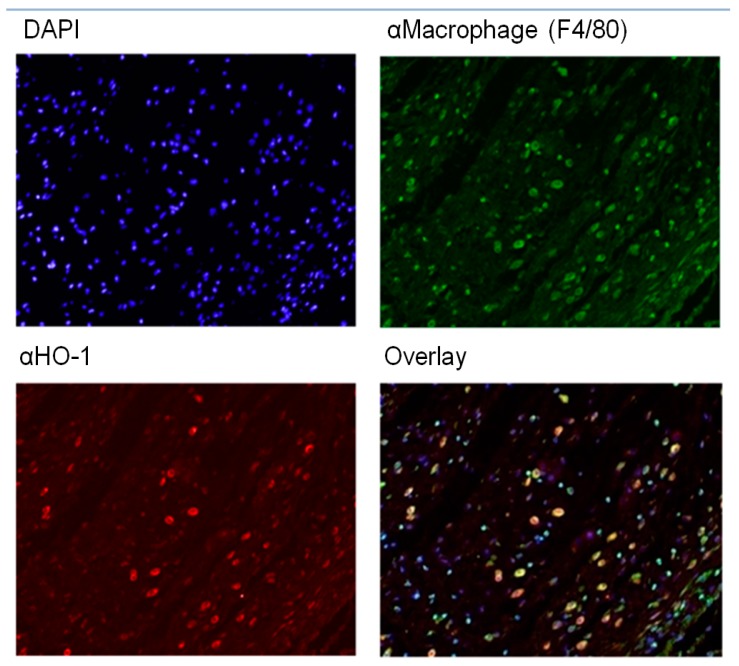
Co-localization of HO-1 and macrophages during excisional wound healing. Fluorescent staining from a previous experiment on excisional wound healing in C57Bl/6 mice at day 2 post wounding, showing cell nuclei stained with DAPI (**blue**), HO-1 (**red**), macrophages stained for F4/80 (**green**), and an overlay. The overlay picture clearly shows co-localization (**orange**) of HO-1 and macrophages.

HO-1 protein was found in the epithelial cells of the epidermis and in recruited leukocytes in the dermis (Figure 3b). In the epidermis, HO-1-positive cells were clustered in the re-epithelialized tissue underneath the wound crust and were likely newly-formed keratinocytes [60,61]. In the dermis, HO-1-positive cells in inflamed tissues were individually spread, and based on their location and morphology, they appear to be macrophages [60,61,62,63]. Moreover, in unpublished data from a previous experiment on excisional wound healing in C57Bl/6 mice at day-2 post-wounding, fluorescent staining for HO-1 (red) and F4/80 (green) clearly showed co-localization (orange) of HO-1 and macrophages in a majority of cells (Figure A2).

The wounds were scored for the levels of HO-1 expression in the epidermal and dermal regions, and compared between the different treatment groups (Figure 3c). RIPC treatment did not modulate HO-1 protein expression in either region of day-7 wounds when compared to controls. Variations in HO-1 expression was found between animals, but was independent of RIPC treatment. In summary, RIPC treatment does not appear to alter HO-1 mRNA and protein expression in day-7 wounds.

### 2.4. Effects of RIPC on Wound Morphology and Collagen Deposition

To determine if RIPC modulates other processes during wound repair, we performed H&E staining to examine wound morphology, and AZAN staining to investigate the effects on collagen deposition.

**Figure 4:**
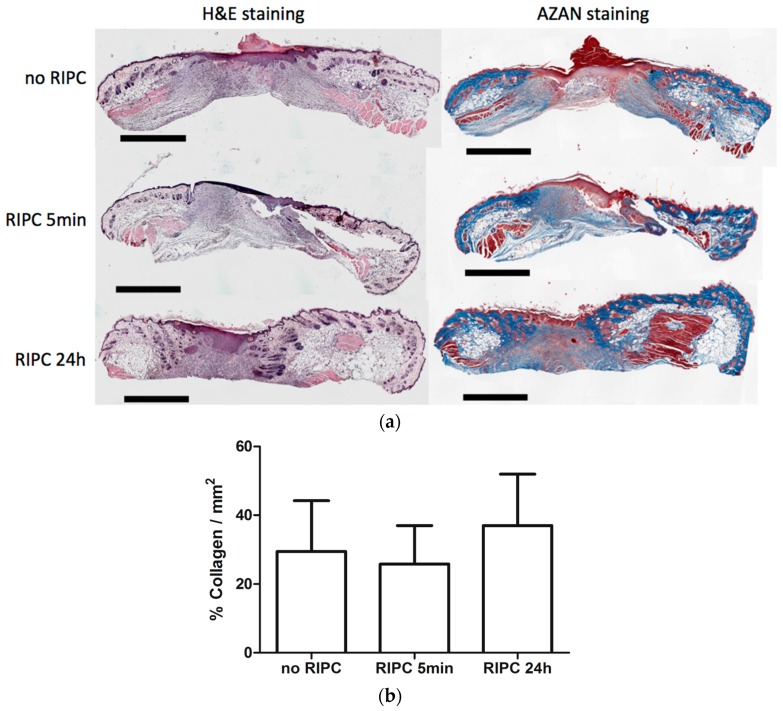
Effects of early and late RIPC on morphology of 7-day-old excisional wounds. (**a**) H&E and AZAN staining were performed to evaluate wound morphology (bars represent 1 mm); (**b**) Quantification of collagen deposition to assess the level of wound remodeling using AZAN staining. Data are expressed as mean ± SD. No significant differences were observed between the different groups: no RIPC (*n* = 8), RIPC 5 min (*n* = 6) and RIPC 24 h (*n* = 6).

H&E staining revealed that the wound area could be easily distinguished from non-injured skin by a disruption of the epidermis, subcutaneous fat, and muscle layers. Figure 4a shows H&E staining of day-7 wounds from mice treated with early and late RIPC, and control mice (left). At the surface of the wound, the re-epithelialized tissue had marked epithelial hyperplasia under the crust of the wound. More distally, the highly cellularized granulation tissue was less organized, and consisted of inflammatory cells, such as macrophages, granulocytes, and (myo)fibroblasts. Variations in the thickness and size of the wounds were observed between the tissue sections of the animals. When comparing the different treatment groups, no differences were found in morphology and in the presence of different cell types in day-7 wounds.

Figure 4a (right) shows collagen deposition by AZAN staining. The wound regions were marked after which the level of collagen deposition in the wounds were measured and corrected for the total wound area (Figure 4b). No significant differences were observed between the different groups. Summarizing, RIPC did not affect wound morphology and collagen deposition of day-7 wounds.

## 3. Discussion

We postulated that RIPC increases HO-1 induction and improves cutaneous wound repair. RIPC induced HO-1 in kidney, heart, and skeletal muscles, but not in the skin. Although RIPC had previously been shown to target the skin [44,45,46,48], both early and late RIPC did not affect cutaneous wound closure. In addition, skin morphology and collagen deposition at day-7 wounds did not change after early or late RIPC.

RIPC-mediated protection to organs seems therefore tissue-specific and/or dependent on the insult. Organ- and time-specific protective effects of RIPC have also previously been demonstrated. For example, RIPC does not improve wound healing in small bowel anastomoses [64,65]. Although late RIPC (24 h) attenuates ischemia/reperfusion injury (IRI) in muscle flaps, it is ineffective in adipocutaneous flaps [66]. In contrast, early RIPC (30 min) enhances adipocutaneous flap survival [67]. IPC improves the survival of myocutaneous and skin flaps subjected to secondary ischemia of 1h in rats [68,69]. Since these RIPC protocols vary from ours, and IPC and RIPC are different procedures, these studies cannot be directly extrapolated to our study. The protective actions of RIPC are thus dependent on the targeted organ and the type of RIPC treatment [70]. Remote preconditioning by trauma (RPCT) by abdominal incision, has previously been reported to improve cardiac outcome following induced heart infarcts by coronary artery occlusion in murine and canine models [71,72,73]. Similarly, the inflicted injuries in our study could have led to the induction of overlapping cytoprotective pathways. When these RPCT-induced protective pathways were stronger than the effects by RIPC or used similar pathways, this could explain in part the observed lack of protective effects by RIPC on cutaneous wound repair. Similar protective pathways of RIPC and RPCT include the activation of protein kinase C, mitogen-activated protein kinases and mitochondrial potassium ATP channels, bradykinin and adenosine [71,73,74].

**Figure A3:**
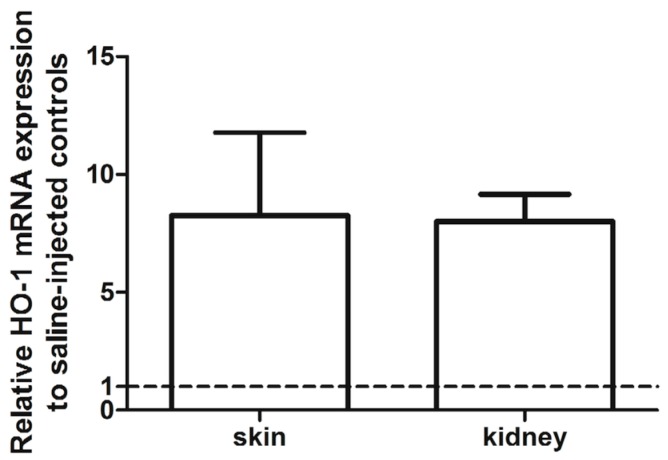
Pharmacological preconditioning with cobalt protoporphyrin (25 mg/kg body weight) in HO-1 *luc* Tg mice (*n* = 6) induced HO-1 mRNA expression in both the skin and kidney 24 h after treatment when compared to saline-injected control mice (*n* = 6). Dashed line represents the mRNA expression levels in the corresponding control organs after saline injection, which are set at 1. Data represents the relative mean ± SD. One significant outlier was found in both skin and kidney group and therefore excluded in the graph.

Previously, the stress enzyme HO-1 was found to be important in wound repair and is generally expressed at wound sites [60,75,76]. HO-1 and HO-2 knockout mice showed a delayed wound repair; whereas, induction of HO-1 or administration of its effector molecule bilirubin accelerated wound repair [19,20,77]. Since some of the protective effects of RIPC were shown to be dependent on HO-1 expression in IRI of diverse organs, like the liver [23,24,26], lungs [33], and intestines [28], we further evaluated the role of RIPC on HO-1 in excisional wound healing. HO-1-*luc* Tg mice allowed monitoring the effects of RIPC on HO-1 promoter activity levels in different organs in real-time. 6 and 24 h after RIPC treatment, HO-1 promoter activity was significantly induced compared to 1h after RIPC. This correlates well with our RT-PCR data showing significantly increased endogenous HO-1 mRNA levels in muscle, heart, and kidney 6h after RIPC treatment. However, despite RIPC improving cutaneous microcirculation [44], no HO-1 mRNA induction was found in the skin, and may thus not affect the skin directly. HO-1 induction in the skin is possible using pharmacological preconditioning since we previously observed that i.p. administration of the HO-1 inducer cobalt protoporphyrin at a concentration of 25 mg/kg body weight in HO-1 *luc* Tg mice induced HO-1 mRNA specifically in the skin after 24 h (Figure A3). This underscores the tissue- and time-dependent effects of RIPC, which is probably due to the structural and physiological differences between different organs. Since organs that have induce HO-1 expression upon RIPC treatment correlate with the organs that are protected by RIPC, it is tempting to speculate that HO-1 facilitates these protective effects.

Also the long-term protective effects of RIPC via activation of HO-1 were absent in the skin. Both HO-1 mRNA and protein expression levels were observed to be independent of RIPC treatment in the epidermal and dermal regions of day-7 wounds. Like others, we found HO-1-positive keratinocytes in the hyperproliferative epithelia of the wound margins covering the wound [60,61,78]. In the dermis, we also observed HO-1-positive infiltrating leukocytes that are likely macrophages [60,62,79]. HO-1-positive macrophages are thought to protect the wound environment against oxidative stress [60,80]. The pro-inflammatory HO substrate heme is abundantly released at the edges of the wound site and stimulates recruitment of leukocytes [60,63,78]. HO-1 is thought to attenuate these inflammatory and oxidative triggers at the wound site.

The effect of both early and late RIPC on wound closure was monitored regularly. However, the (immuno)histochemical and PCR analysis was only performed on day 7 wounds, which limits our insight in wound repair processes, like inflammatory signaling, during the first days. Using the HO-1-*luc* Tg mice we previously found that HO-1 promoter activity was indeed significantly induced on day 3 post-wounding, however the level of HO-1 promoter activity did not decline significantly at day 7 compared to day 3 [79]. Although no effects in wound closure, collagen deposition, or HO-1 expression in the skin were observed, we cannot exclude that paracrine effects of HO-1 or other protective signaling pathways may have been triggered by RIPC. HO-effector molecules biliverdin, bilirubin, CO, and ferritin, have all shown to improve wound repair [20,51,52,53,54], suggesting that RIPC-induced HO-1 induction in various organs stimulate wound repair in a paracrine fashion. Interestingly, increased systemic levels of bilirubin augment vascular function [81,82] and may contribute to the reported RIPC induced improvement of cutaneous microcirculation. In a previous study, no adverse effects of HO inhibition following RIPC were observed in a kidney injury model, suggesting that other mediators may have been protective [31]. Alternative protective pathways triggered by RIPC include humoral, neural, or systemic anti-inflammatory, anti-apoptotic responses [22,59]. In addition, RIPC may have more effects in more stringent wound repair models where there is a shortage of cytoprotective molecules, such as in diabetic wound repair models and pressure ulcers [47]. Although our method has shown to be effective in diverse animal models [32], other RIPC regimens may enhance these protective effects such as combinations with remote ischemic postconditioning [35,36,47]. Recently, it was found that the sex of the animal may play a role in the efficacy of RIPC treatment, and was observed to be lower in experimental groups of mixed sexes, which we also used in our wound repair study [83].

## 4. Materials and Methods

### 4.1. Animals

The Committee for Animal Experiments of the Radboud University Nijmegen approved all procedures involving animals (RU-DEC 2010-248) on 1 February 2011. Fifty mice (strain: HO-1-*luc* FVB/N-Tg background; see Table 1) of 4–5 months in age, and weighing 21–35 g were provided with food and water ad libitum and maintained on a 12 h light/dark cycle and specific pathogen-free housing conditions at the Central Animal Facility Nijmegen. The transgene consists of the full-length mouse HO-1 promoter fused to the reporter gene luciferase (*luc*). More details on the housing conditions are previously described [84]. Mice were originally derived from Stanford University (Stanford, CA, USA) as previously described [85]. An overview of the animals used for the different experiments can be found in Table 1. No animals died during the experiments and no animals were excluded during the experiments or data analyses. All mice were randomly divided over the experiments, and split evenly over their sex and age. All outcomes were measured by an observer who was blinded for the allocation of the animals to the experimental groups, when possible.

### 4.2. RIPC Treatment

RIPC by brief hind limb ischemia was induced by applying elastic latex-free O-rings (Miltex Integra: 28–155) using a hemorrhoidal ligator (Miltex McGivney: 26–154B) bilaterally around the most upper position of the proximal thigh (Figure A1). Reperfusion was accomplished by cutting the elastic rings with scissors, confirmed by the disappearance of blue color to the limbs (Figure A1) as described previously [86,87,88]. The mice were anesthetized with isoflurane in O_2_/N_2_O (5% isoflurane for induction and 2%–3% to maintain anesthesia) during RIPC treatment and treatment consisted of three cycles of 4-min ischemia interspersed with 4-min reperfusion. This RIPC regime is based on a previous study in which we found that bilateral repetitive (3 times 4 min) ischemia/reperfusion gave the most potent protection in a kidney injury model [32].

### 4.3. Measuring of HO-1 Promoter Activity

In order to monitor HO-1 promoter activity after RIPC treatment in time, HO-1-*luc* Tg mice underwent RIPC treatment as described above. HO-1-*luc* expression was measured in vivo and the mice were sacrificed at 24 h. In vivo bioluminescence imaging was performed as described before on the IVIS Lumina System (Caliper Life Sciences, Hopkinton, MA, USA) [89]. Images taken were quantified using Living Image 3.0 software (Caliper Life Sciences) by selecting the regions of interest (ROI). ROIs included both the dorsal images encompassing the back region below the head and above the tail to cover the area where the wounds would be made and the renal area. Emitted photons per second (or total flux) per region of interest (ROI) was measured, and then calculated as fold change from baseline levels and related to 1 h after RIPC.

### 4.4. Excisional Wound Model

Wounds were made 24 h or 5 min after RIPC. Control mice did not receive RIPC, but underwent the same anesthetic procedure 1h before wounding. Two full-thickness excisional wounds of 4 mm in diameter were made on the shaved dorsal side of anesthetized mice using a sterile disposable biopsy punch (Kai Medical, Seki City, Japan), as previously described by our group [77]. The wounds were created on the dorsum to either side of the midline, with approximately 1cm between the wounds, and just below the shoulders and pelvis. Skin biopsies taken to create the 4-mm wounds served as control skin. Wounds were photographically documented immediately, and 1, 3 and 7 days after wounding with a ruler placed perpendicular to the wounds for wound size normalization. The area of the wounds was blindly measured twice using ImageJ v1.44p software (http://imagej.nih.gov/ij; NIH, Bethesda, MD, USA).

### 4.5. Sample Collection

At day 7, the mice were anesthetized with 5% isoflurane in O_2_/N_2_O and sacrificed by exsanguination, followed by cervical dislocation. Kidney and muscle (*m. quadriceps femoris*) were dissected, and wound tissue was collected using a 4-mm biopsy punch. Half of the tissue was fixated with 4% paraformaldehyde and processed for paraffin embedding and (immuno)histochemistry, and the other half was snap frozen in liquid nitrogen and stored at −80 °C until use for RT-PCR.

### 4.6. (Immuno-)histochemical Staining and Analyses

Standard H&E, Weigert-AZAN staining (azo carmine and aniline blue), and immunohistochemical HO-1 staining were performed on paraffin sections of the wounds as previously described [77]. Stained sections were analyzed and photographed using the Zeiss Imager Z1 microscope (Zeiss, Sliedrecht, The Netherlands) and Axiovision software version 4.8 (Zeiss).

Analysis of collagen deposition in AZAN stained wound sections was performed by image analysis using a macro built in Image J [90]. The wound area was manually defined using the edges of the *panniculus carnosus* and epithelium as boundaries before running the macro. Measurements were averaged per mouse and mean intensity/mm^2^ was used for further analysis.

HO-1 immunoreactivity was evaluated by blindly scoring the epidermal zone and the dermal region of the wounds. A single section per wound of each animal was semi-quantitatively scored as previously described using the following scale: 0 (minimal), 1 (mild), 2 (moderate), and 3 (marked).

### 4.7. RNA Isolation and Quantitative-RT-PCR

Tissue was pulverized in TRIzol (Invitrogen, Carlsbad, CA, USA) using a micro-dismembrator (Sartorius BBI Systems GmbH, Melsungen, Germany) and RNA was further extracted as previously described [13]. All values were normalized to the household gene *gapdh*, which is often used in RIPC experiments [91,92] according to the comparative method (2^−ΔΔ*C*t^). *Gapdh* mRNA expression levels were stable and were not affected by RIPC treatment. The sequences of the mouse-specific primers for *gapdh* are forward 5′GGCAAATTCAACGGCACA3′, and reverse 5′GTTAGTGGGGTCTCGCTCCTG3′, and for *Hmox1* (*HO-1*) forward 5′CAACATTGAGCTGTTTGAGG3′, and reverse 5′TGGTCTTTGTGTTCCTCTGTC3′.

### 4.8. Statistics

Data were analyzed using GraphPad Prism 5.01 software (San Diego, CA, USA). Outliers were tested using the Grubbs’ test, but no outliers were found (except for the data in Figure A3 where one outlier was found in the skin and one in the kidney group). Data were analyzed using two-sided *t*-tests to compare two variables or a one-way analysis of variance when comparing multiple variables. Bonferroni’s multiple comparison *post hoc* test was applied as correction for multiple comparisons when investigating multiple dependent research questions. Results were considered significantly different at *p* < 0.05 (* *p* < 0.05, ** *p* < 0.01, and *** *p* < 0.001).

## 5. Conclusions

RIPC treatment induced HO-1 mRNA expression in kidney, heart, and ligated muscle, and may therefore directly contribute to enhanced protection to injurious stressors and/or microcirculation in these tissues. However, RIPC did not alter HO-1 in the skin and was not modulated in day-7 skin wounds, demonstrating organ- and time-specific effects. Both early and late RIPC treatment did not affect dermal wound closure time, collagen deposition, or wound morphology. A better understanding of the mechanistic insight by which RIPC mediates organ protection is needed. 

## Figures and Tables

**Table 1:** Overview animal experiments.

Aim Experiment	Read Out	Animals (*n*: ♂/♀)
Investigate the effects of RIPC on HO-1 promoter activity	HO-1 promoter activity at 1, 6 and 24 h after RIPC treatment	6: 0/6
Investigate the effects of RIPC on *HO-1* gene expression in different organs during time	HO-1 mRNA levels at 0, 1, 6, and 24 h after RIPC treatment	24: 0/24 (6 per time point)
Investigate the effects of RIPC on dermal wound healing	Early (5 min before wounding) and late (24 h before wounding) effects of RIPC on wound healing compared to controls without receiving RIPC treatment (endpoint: day 7)	6: 4/2 (early RIPC) 6: 4/2 (late RIPC) 8: 4/4 (controls)

## Abbreviations

H&E	hematoxylin and eosin
HO-1	heme oxygenase-1
HO-1-*luc* Tg	HO-1-*luciferase* transgenic
I/R	Ischemia/Reperfusion
IPC	Ischemic preconditioning
IRI	Ischemia/Reperfusion injury
RIPC	Remote ischemic preconditioning
RPCT	Remote preconditioning by trauma
ROS	Reactive oxygen species
